# Unrecognized High Occurrence of Genetically Confirmed Hereditary Carnitine Palmitoyltransferase II Deficiency in an Austrian Family Points to the Ongoing Underdiagnosis of the Disease

**DOI:** 10.3389/fgene.2019.00497

**Published:** 2019-05-22

**Authors:** Christina Zach, Karl Unterkofler, Peter Fraunberger, Heinz Drexel, Axel Muendlein

**Affiliations:** ^1^Vorarlberg Institute for Vascular Investigation and Treatment, Feldkirch, Austria; ^2^Medical Central Laboratories, Feldkirch, Austria; ^3^Breath Research Institute, University of Innsbruck, Dornbirn, Austria; ^4^Vorarlberg University of Applied Sciences, Dornbirn, Austria; ^5^Private University of the Principality of Liechtenstein, Triesen, Liechtenstein; ^6^Division of Angiology, Swiss Cardiovascular Center, University Hospital of Bern, Bern, Switzerland; ^7^Drexel University College of Medicine, Philadelphia, PA, United States

**Keywords:** carnitine palmitoyltransferase II deficiency, family study, mutation, underdiagnosis, pedigree

## Abstract

Adult muscle carnitine palmitoyltransferase (CPT) II deficiency is a rare autosomal recessive disorder of long-chain fatty acid metabolism. It is typically associated with recurrent episodes of exercise-induced rhabdomyolysis and myoglobinuria, in most cases caused by a c.338C > T mutation in the *CPT2* gene. Here we present the pedigree of one of the largest family studies of CPT II deficiency caused by the c.338C > T mutation, documented so far. The pedigree comprises 24 blood relatives of the index patient, a 32 year old female with genetically proven CPT II deficiency. In total, the mutation was detected in 20 family members, among them five homozygotes and 15 heterozygotes. Among all homozygotes, first symptoms of CPT II deficiency occurred during childhood. Additionally, two already deceased relatives of the index patient were carriers of at least one copy of the genetic variant, revealing a remarkably high prevalence of the c.338C > T mutation within the tested family. Beside the index patient, only one individual had been diagnosed with CPT II deficiency prior to this study and three cases of CPT II deficiency were newly detected by this family study, pointing to a general underdiagnosis of the disease. Therefore, this study emphasizes the need to raise awareness of CPT II deficiency for correct diagnosis and accurate management of the disease.

## Introduction

Degradation of fatty acids via mitochondrial β-oxidation is an essential pathway for energy homeostasis, especially in situations with high energy demand and glucose limitation, such as prolonged fasting or exercise. For β-oxidation to occur, the long-chain fatty acids have to be transported across the mitochondrial membrane by the carnitine palmitoyltransferase (CPT) system, consisting of CPT I and II, located in the outer (CPT I) and inner mitochondrial membrane (CPT II). Defects of these enzymes, such as CPT II deficiency, disrupt β-oxidation and lead to disorders of fatty acid metabolism (Bonnefont et al., 2004; Houten and Wanders, 2010; Lehmann et al., 2017).

Carnitine palmitoyltransferase II deficiency is a rare autosomal recessive hereditary disorder with three clinical phenotypes: a lethal neonatal form, a severe infantile and a mild adult myopathic form (Bonnefont et al., 2004; Lehmann et al., 2017). Muscle CPT II deficiency in young adults is the most frequent type. Patients present primarily with recurrent attacks of myalgia and muscle weakness, associated with rhabdomyolysis and myoglobinuria (Deschauer et al., 2005; Joshi et al., 2018). Treatment includes avoidance of known triggers such as fasting and excessive exercise, in addition to a low-fat/high carbohydrate diet and in some cases L-carnitine administration (Wieser, 2017).

Diagnosis of CPT II deficiency is commonly confirmed by sequence analysis of the *CPT2* gene. The most frequent *CPT2* pathogenic mutation in Caucasians is the missense variant c.338C > T (p.Ser113Leu, rs74315294) found in up to 90% of symptomatic patients (Joshi et al., 2014), but only in 0.1 – 0.2% of the general European population (Sherry et al., 2001; Lek et al., 2016). Around 60% –70% of the patients with CPT II deficiency are homozygous for the c.338C > T mutation and remaining others are compound heterozygotes, many of which are carrying the common c.338C > T variant in combination with various rarely occurring mutations of the *CPT2* gene (Isackson et al., 2006; Corti et al., 2008; Joshi et al., 2014).

Despite the knowledge of the genetic background and advances made in non-invasive diagnosis of the disease, CPT II deficiency is still underdiagnosed, probably due to variable degrees of the symptomatology as well as lack of awareness among the general public and health care professionals (Balasubramanian et al., 2018).

Here we present the pedigree of one of the largest family studies of CPT II deficiency caused by the c.338C > T mutation documented so far. It includes 24 blood relatives of the index patient, over three generations and shows a higher than expected prevalence of this rare hereditary disease within the tested family.

## Case Presentation

The index patient of this family study is a 32 year old female from Austria, who presented with a long standing history of myalgias and recurrent, exercise-induced rhabdomyolysis episodes accompanied by myoglobinuria. First symptoms occurred in childhood, around 5 years of age. After prolonged physical activity such as hiking or skiing she experienced severe myalgias and muscle weakness combined with dark colored urine. At this point, however, she did not seek medical attention and the episodes self-resolved. Several years later she moved to Australia, where she was admitted at the age of 32, to the acute medical unit of the Royal Adelaide Hospital (Australia) due to severe whole-body rhabdomyolysis with myoglobinuria. Her creatine kinase (CK) level was significantly raised to 45,560 U/L, but her kidney function was normal. She was treated with an intravenous fluid therapy and was kept in the hospital for the following 5 days, where her muscles aches gradually improved and her CK levels decreased. In her family history she reported that her maternal uncles were also affected by recurrent episodes of rhabdomyolysis and myoglobinuria.

A subsequent mutation analysis of the *CPT2* gene using a custom 1,037 gene Roche 1 k Disease (R1kD) Seq Cap EZ capture kit on a NextSeq^®^ Sequencing System instrument (Illumina, San Diego, CA, United States), revealed a homozygous c.338C > T mutation, confirming the diagnosis of the suspected adult muscular form of hereditary CPT II deficiency.

**FIGURE 1:**
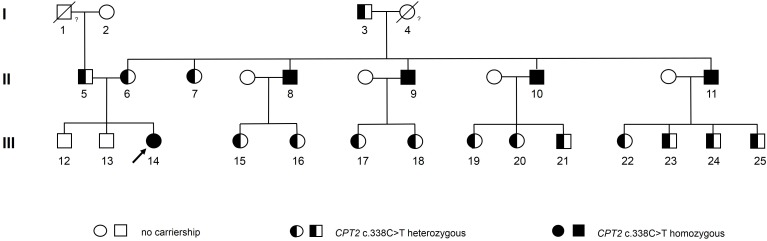
Pedigree of an Austrian family with frequent occurrence of CPT II deficiency caused by the *CPT2* c.338C > T mutation. The index patient is marked with an arrow. Genotypes of the deceased participants 1 and 4 may be assumed as heterozygous and homozygous, respectively, based on reported symptoms and statistical considerations (as given in the Supplementary Material).

Thereupon, the Austrian family members of the index patient were invited to take part in this family case study. All participants gave written informed consent for genetic testing of the *CPT2* c.338C > T variant as well as for their participation in the study and for publication of this case report. Genotyping analysis was performed by Sanger sequencing as described in the Supplementary Material. The pedigree of the index patient together with results from genotyping is given in Figure 1. Sequence analysis showed that all four maternal uncles of the index patient were homozygous for the *CPT2* c.338C > T allele (participants 8, 9, 10, and 11 in the pedigree). Information on age at which symptoms first appeared, hospitalization due to rhabdomyolysis, nutrition, and sports activities was obtained by a standardized questionnaire.

All four maternal homozygous uncles aged between 57 and 61 years reported recurrent events of exercise-induced myalgia and rhabdomyolysis of various degrees, with first symptoms occurring during childhood, between five and eight years of age. Prior to this study, for three of them (participant 8, 10, and 11 in the pedigree) severe whole-body rhabdomyolysis accompanied by myoglobinuria, had led to hospitalization, at least once. Tandem mass spectrometry analysis of a muscle biopsy sample of participant 8 showed elevated long-chain acylcarnitine levels (C16 and C18:1), associated with CPT II deficiency. Furthermore, enzyme activity of CPT II in leukocytes was only at 3% of the control value, confirming diagnosis of CPT II deficiency for participant 8. Although a metabolic myopathy was suspected for participants 10 and 11, no further tests were performed and CPT II deficiency remained undiagnosed.

Moreover, one homozygous maternal uncle (participant 9) never sought medical attention, even though he experienced several episodes of rhabdomyolysis and myoglobinuria throughout his life.

Furthermore, we identified 15 family members as heterozygous carriers of the genetic variant (see pedigree). None of them, however, reported any clinical symptoms indicating CPT II deficiency.

The maternal grandmother of the index patient (#4 in the pedigree) died prior to this study and could, therefore, not be genetically tested. However, based on the established genotypes of her children multinomial distribution revealed that the odds for a homozygous genotype are 16 times higher as for the heterozygous genotype (statistical considerations are described in detail in the Supplementary Material). As for the deceased asymptotic paternal grandfather of the index patient (#1 in the pedigree) we assumed a heterozygous carriership to be most likely.

## Discussion

Here we reported one of the largest family studies of CPT II deficiency documented so far. The pedigree presented in this study comprises 24 blood relatives of the index patient over three generations, with 20 affected individuals including five homozygous and 15 heterozygous carriers of the c.338C > T mutation. Additionally, two already deceased relatives of the index patient were carriers of at least one copy of the genetic variant.

Prior to this study, all five living homozygous family members had experienced recurrent rhabdomyolysis events that had led to hospitalization for four of them. However, beside the index patient only one of them (participant 8) had been correctly diagnosed with CPT II deficiency. Although two patients (10 and 11) sought medical care due to recurrent episodes of myoglobinuria, CPT II deficiency remained undiagnosed. Here, it should be noted that several established as well as hospital physicians were involved in the care of these patients pointing to the low awareness of the disease, even in countries with high medical standards, like Austria. Underdiagnosis such as this, is a known problem of the condition (Balasubramanian et al., 2018; Gjorgjievski et al., 2018). Many patients attribute myalgia or muscle weakness after physical activity to lack of physical fitness and do not seek medical advice, even when they experience myoglobinuria events. Moreover, the condition often remains underdiagnosed due to lack of understanding of its clinical representation (Wieser et al., 2003).

Correct diagnosis of CPT II deficiency, however, is crucial to prevent recurring episodes and set appropriate measures to avert grave complications such as acute kidney injury, should rhabdomyolysis and myoglobinuria occur. Typically, heterozygous carriers do not show a clinical phenotype, though cases of heterozygous carriers with mild to severe symptoms are known (Fanin et al., 2012; Joshi et al., 2012). In this respect, primarily the progenies of heterozygous carries are of clinical relevance, since they are at risk of becoming symptomatic homozygotes or compound heterozygotes with a more serious form of the disease (Taggart et al., 1999; Thuillier et al., 2000; Vladutiu et al., 2002). However, genetic testing for the CPT2 c.338C > T mutation in asymptomatic subjects is not recommended. Detecting heterozygous carriers could cause unwarranted social problems such as discrimination against hereditary illness and/or refusal of acceptance for health insurance. More importantly, psychological stress could be increased when muscle symptoms, which are probably not caused by genetic CPT II deficiency, occur.

On the maternal side of the index patient the number of individuals affected by the c.338C > T mutation is remarkably high. In fact, both maternal grandparents showed a heterozygous or a most likely homozygous genotype. The affected grandparents (#3 and #4 in the pedigree) on the maternal side both originate from the same remote Austrian village. Elevated appearance of homozygous or heterozygous individuals of rare hereditary diseases within a secluded population is a well-known phenomenon. Over past centuries geographic isolation of secluded communities restricted the genetic exchange and thereby favored the propagation of rare autosomal recessive diseases (Zlotogora, 2007). Further insight into the local frequency of the disease could be gained by investigating the prevalence of CPT II deficiency in other families originating from the same area. Particularly with regard to the general underdiagnosis of the disease the identification of symptomatic homozygous affected patients would contribute to appropriate disease management and prevention of future serious episodes.

Notably, the heterozygous father and the heterozygous mother of the index patient (#5 and #6 in the pedigree, respectively) originate from other regions in Austria and belong to different religion confessions. Therefore, any relationship between the father and the mother of the index patient appears unlikely. The high prevalence of the CPT2 mutation within the family of the index patient, even in unrelated family members, may point to a higher frequency of the CPT2 c.338C > T mutation in certain Middle European areas than given in public mutation databases for a Caucasian population (Sherry et al., 2001; Lek et al., 2016). This issue may be clarified in future genome projects.

In conclusion, our study showed that CPT II deficiency is generally underdiagnosed. Furthermore, the c.338C > T mutation might have a higher prevalence than stated by genetic databases, especially in populations with limited genetic exchange. Therefore, this study emphasizes the need to expand the awareness of CPT II deficiency in the general public and health care professionals for correct diagnosis and accurate management of the disease.

## Data Availability

The raw data supporting the conclusions of this manuscript will be made available by the authors, without undue reservation, to any qualified researcher.

## Ethics Statement

All participants gave written informed consent for CPT2 mutation testing. As stated by the Ethics Committee of the Land Vorarlberg, Austria, no ethical approval is required for the present investigation.

## Author Contributions

CZ and AM wrote the manuscript. KU and AM designed the study and interpreted the data. KU was responsible for patient recruitment and provided the patient data. HD and PF contributed to design, discussion, and edited the manuscript.

## Conflict of Interest Statement

The authors declare that the research was conducted in the absence of any commercial or financial relationships that could be construed as a potential conflict of interest.
