# Supplementary material for: Unrecognized High Occurrence of Genetically Confirmed Hereditary Carnitine Palmitoyltransferase II Deficiency in an Austrian Family Points to the Ongoing Underdiagnosis of the Disease
Source: Front Genet. 2019 May 22;10:497. doi: 10.3389/fgene.2019.00497 (PMC6540962; doi:10.3389/fgene.2019.00497)
Supplement: Supplementary file 1 [file Data_Sheet_1.PDF]

Supplementary Material

1 Genotyping of the CPT2 C.338C>T mutation

Cells for DNA-preparation were collected using buccal swabs. Genomic DNA was extracted from buccal swabs by standardized techniques using the QIAamp DNA Mini Kit (Qiagen, Hilden, Germany) according to manufacturer`s instructions. A pair of PCR primers was designed flanking the *CPT2* c.338C>T variant and tagged with M13 forward and M13-40 universal primers to be used in the subsequent sequencing reactions. Primer sequences were as follows (M13 forward and M13-40 universal primers age given as lower case letters): Forward primer: tgtaaaacgacggccagCTATTATGAGTTCCTCGCCATGA; reverse primer: gttttcccagtcacgacCGTTACTTCATTTGCTGGTCTCAC. PCR was performed under the following amplification conditions: 5 min at 95°C, then 35 cycles at 95°C for 20 s, 55°C for 30 s, and 72°C for 30 s, followed by a final extension step of 72°C for 5 min. PCR-products were purified using the ExoProStar™ 1-STEP Kit (GE Healthcare, Chicago, IL, USA) and sequenced using the BigDye® Terminator v3.1 Cycle Sequencing Kit (Thermo Fisher Scientific, Waltham, MA, USA) on a 3730 DNA Analyzer (Thermo Fisher Scientific).

2 Statistical considerations

(i) The father of the index patient (#5 in the pedigree) was tested heterozygous for the *CPT2* c.338*T allele. As the father's mother (#2 in the pedigree) was tested wild-type it follows that his father (#1 in the pedigree, already deceased) was either homozygous or heterozygous. The latter being more likely since symptoms of CPT II deficiency have never been reported though he was physically very active.

(ii) The mother of the index patient is one of the two sisters (#6 and #7 in the pedigree) who were tested heterozygous. Her four brothers (#8 - #11 in the pedigree) were all tested homozygous. As their father (#3 in the pedigree) was tested heterozygous it follows that their mother (#4 in the pedigree, already deceased) was either homozygous or heterozygous.

As usual we will denote the probability of an event A occuring by $P(A)$ and the conditional probability of the event A occurring given that event B has occurred by $P(A|B)$.

Now we define the following events: K is the event that 4 of 6 children are homozygous and 2 are heterozygous. E is the event that both of their parents are heterozygous. $\bar{E}$ is the event that this is not the case. The latter is equivalent to the statement that the maternal grandmother of the index patient was homozygous, since the maternal grandfather is known to be heterozygous.

To calculate the probability $P(K|E)$ (that is the probability that 2 of 6 children are heterozygous and 4 children are homozygous under the assumption that both parents are heterozygous) we apply the multinomial distribution formula for the three possible outcomes: number of children which are wild-type x_1, number of children which are heterozygous x_2, number of children which are homozygous x_3, occurring with the corresponding probabilities p_1, p_2, and p_3.

$$P(X_1 = x_1, X_2 = x_2, X_3 = x_3) = \frac{(x_1 + x_2 + x_3)!}{x_1!x_2!x_3!} p_1^{x_1} p_2^{x_2} p_3^{x_3}.$$

Inserting the numbers in the equation yields:

$$P(K|E) = P(X_1 = 0, X_2 = 2, X_3 = 4) = \frac{6!}{0!2!4!}\left(\frac{1}{4}\right)^0 \left(\frac{1}{2}\right)^2 \left(\frac{1}{4}\right)^4 = \frac{15}{1024}.$$

Now we calculate the probability $P(K|\bar{E})$ (that is the probability that 2 of 6 children are heterozygous and 4 children are homozygous under the assumption that the maternal grandmother was homozygous):

$$P(K|\bar{E}) = P(X_1 = 0, X_2 = 2, X_3 = 4) = \frac{6!}{0!2!4!}\left(\frac{1}{2}\right)^2 \left(\frac{1}{2}\right)^4 = \frac{15}{64}.$$

From the calculations of these two probabilities we conclude that the event K is 16 times more likely to appear if the maternal grandmother was homozygous. But does this mean that she really is more likely to have been homozygous? To calculate the probability that the maternal grandmother was homozygous, i.e., the probability $P(\bar{E}|K)$ one would have to apply Bayes' theorem but the sample size of 6 children is insufficient to draw a definite conclusion.
